# P02.144. Evaluation of health outcomes for members of a managed care organization referred for acupuncture

**DOI:** 10.1186/1472-6882-12-S1-P200

**Published:** 2012-06-12

**Authors:** E Sommers, J Kailey

**Affiliations:** 1 Pathways to Wellness, Boston, USA; 2 Boston University School of Public Health, Boston, USA

## Purpose

A community health center and clinic specializing in acupuncture partnered with a large managed care organization to determine whether acupuncture treatment might influence clinical outcomes and costs of care. Members of the managed care organization were referred through their physicians to receive up to 20 treatments for any of the following conditions: pain, headache, menstrual or menopausal symptoms, and carpal tunnel syndrome. Although the project is ongoing, results of the first 4 years of the project will be presented.

## Methods

Data for this observational assessment were collected on patient demographics, health history, clinical outcomes, and associated costs of care. All patients received individualized acupuncture treatments provided by licensed acupuncturists according to standards of care.

## Results

Data were collected on 307 individuals who were referred for acupuncture treatment. Eighty percent were female, 45% were Hispanic/Latino, 10% were African-American, and the mean age was 38 years. Two hundred sixty-six individuals received one or more acupuncture treatments. Of these, 71% were referred for pain and 18% were referred for headache. Fewer members were referred for menstrual (13), menopausal (6), or carpal tunnel symptoms (7). Mean reduction in pain based on a 10-point Likert scale was -2.3 (p<0.0001) and mean reduction in days/month affected by pain was 12.8 (p<0.0001). Duration of painful episodes decreased (p=0.0003) and quality of life improved as indicated by the SF1 (p<0.0001). Headache intensity levels were significantly reduced (p<0.0002) as was headache frequency (p=0.03). Preliminary cost estimates suggest a decrease in other healthcare costs ($40/member/month) and an increased likelihood that individuals who received acupuncture will continue their membership in the managed care plan.

## Conclusion

Preliminary results indicate that offering acupuncture in a community health setting is acceptable and desirable by patients and physicians. Favorable clinical and cost-of-care outcomes were observed in this ongoing project.

